# Zinc Nutrition Responses to Agronomic and Yield Traits, Kernel Quality, and Pollen Viability in Rice (*Oryza sativa* L.)

**DOI:** 10.3389/fpls.2022.791066

**Published:** 2022-05-09

**Authors:** Essam E. Kandil, Aly A. A. El-Banna, Dalia M. M. Tabl, Marwa I. Mackled, Rehab Y. Ghareeb, Asma A. Al-Huqail, Hayssam M. Ali, Jebril Jebril, Nader R. Abdelsalam

**Affiliations:** ^1^Department of Plant Protection, Faculty of Agriculture (Saba Basha), Alexandria University, Alexandria, Egypt; ^2^Rice Research Technology Center (RTTC), Field Crops Research Institute, Agricultural Research Center, Alexandria, Egypt; ^3^Department of Stored Product Pests, Plant Protection Institute, Agriculture Research Center (ARC), Alexandria, Egypt; ^4^Plant Protection and Biomolecular Diagnosis Department, Arid Lands Cultivation Research Institute, The City of Scientific Research and Technological Applications, New Borg El Arab, Egypt; ^5^Chair of Climate Change, Environmental Development and Vegetation Cover, Department of Botany and Microbiology, College of Science, King Saud University, Riyadh, Saudi Arabia; ^6^Department of Agronomy, Kansas State University, Manhattan, KS, United States; ^7^Agricultural Botany Department, Faculty of Agriculture (Saba Basha), Alexandria University, Alexandria, Egypt

**Keywords:** cultivars, fertilization, rice, toxicity, pollen, yield

## Abstract

Rice (*Oryza sativa* L.) is one of the major cereal crops worldwide with wheat and maize. A total of two field experiments were performed to evaluate the response of some rice cultivars to various foliar zinc (Zn) concentrations based on different measurements, such as agronomic, yield, yield compounds, and grain technological parameters. The experimental layout was a split plot in three replicates; the five rice cultivars (Skaha 101, Giza178, Yasmeen, Fourate, and Amber 33) were distributed in the main plots while the four foliar applications of Zn (1,500, 2,000, 2,500 mg/L besides spray water) were occupied the sub-plots. The findings showed significant differences among the five rice cultivars regarding plant height, grain yield, straw yield, biological yield, harvest index, 1,000-grain weight, panicle length, protein percentage, and grain Zn content. There is a significant effect of Zn on all plant attributes. A significant interaction between rice cultivars and foliar application of Zn was observed, whereas fertilizing Giza 178 with foliar application of Zn at the rate of 2,500 mg/L achieved the highest mean values of grain yield and straw yield, biological yield, harvest index, 1,000-grain weight, panicle length, protein %, and Zn content followed by Sakha 101 with Zn application at the rate of 2,000 mg/L, respectively, in both seasons. The rice cultivars significantly differed in hulling (%), broken (%), hardness, grain length, shape, amylose (%), gel consistency, and gelatinization temperature. Unfortunately, the commercial Zn product used was genotoxic to pollen grains with a higher rate of Zn. Aberrations were observed such as stickiness, ultrastructural changes in the exterior and interior walls, partially or fully degenerated grains, and shrunken and unfilled grains. This study concluded that using Zn application at the rate of 2,000 mg/L to protect human and environmental health, the side effects and toxicity of the local commercial Zn product market should be investigated before making recommendations to farmers.

## Introduction

Zinc is a vital micronutrient required for each living organism and plays several essential roles in life, growth, and development; it shows an essential role in the normal physiological activities of growth and development (Fageria et al., 2002; Stanton et al., 2022; Wu et al., 2022). Zn is an essential trace metal with structural roles in controlling proteins, as an enzyme cofactor (Sobczyk and Gaunt, 2022), and it performs as a cofactor for several enzymes involved in numerous processes such as DNA replication, protein synthesis, and metabolism of lipids (Palmgren et al., 2008; Khan et al., 2022). In addition, Zn plays a major role in numerous plant metabolic processes, such as enzyme activity, photosynthesis, chlorophyll synthesis, and other biochemical functions (Arif et al., 2016; Grüngreiff et al., 2020). Zn isthe second highly abundant trace element necessary for all living organisms. It occurs as a divalent cation (Zn^2+^) and is not redox active under physiological conditions, which explains the Zn performance in different physiological roles in a range of biological activities (Kambe et al., 2015).

Zn provides resistance to infections in plants by altering the morphology and physiology of the host plant. One of the most vital roles of micronutrients in plants is their association with a variety of enzyme systems that are engaged in the plant's defense mechanism against pathogens. Zn is also involved in a variety of metabolic activities in plants. Carbonic anhydrase, an enzyme that regulates the conversion of carbon dioxide to reactive bicarbonate species for fixation to carbohydrates in these plants, is modified and/or regulated by Zn. Furthermore, Zn is a component of several other enzymes, including superoxide dismutase and catalase, which protect plant cells from oxidative stress (Shehata et al., 2009).

Zn affects plant water relations, alters stomatal conductance, and plays a role in protecting the cell from the damaging effect of reactive oxygen species (Cakmak, 2008; White et al., 2009; Ghazi et al., 2022). Zn is mainly a plant micronutrient that is involved in several physiological functions; its insufficient supply will decrease the crop yields, their deficiency cause a micronutrient deficiency problem, and nearly, all crops and calcareous, sandy soils, and soils with high phosphorus and silicon become deficient (Rudani et al., 2018). Furthermore, Zn is a vital micronutrient in all biological systems, where it is observed in its oxidized form as a divalent cation (Zn^2+^) (Andreini et al., 2006). The significant limit for Zn differs from 0.38 to 2 μg/g soil based on the crop and soil type (Rahman et al., 2022).

Foliar spray of Zn is more effective than root application due to its higher surface area application, allowing nutrients to be more efficiently absorbed (Fageria et al., 2009; La Bella et al., 2021). It can also be used to satisfy an acute need for macro- and micronutrients. Moreover, some fertilization problems can only be solved by foliar spray application. Micronutrients directly play roles in rice plant production, plant growth, and yield attributes (Jha, 2019). The application of macro- and micronutrients, such as Zn, might not complete the crop requirement for root growth (Timsina and Connor, 2001; Fageria et al., 2009).

Foliage Zn spray as a foliar application on rice seedlings 3 weeks after transplanting was the most effective post-transplanting treatment to recover Zn deficiency (Kumar et al., 1997). The foliar spray could be used effectively to overcome the problem of micronutrient deficiency in subsoil (Torun et al., 2001). Zn application by different methods significantly increased the number of tillers, panicles, plant height, 1,000-grain weight, filled grains%, and grain yield of Sakha 104. Among the different Zn applications, soil application of 15 kg ha^−1^ as ZnSO_4_.H_2_O caused the highest increase in total N percentage, total K percentage, and available Zn content in both grain and straw; however, the percentage of total P decreased significantly. Zinc content in the soil after harvesting was significantly affected by Zn application (Ghoneim, 2016).

Population expansion, increased prosperity, and changing dietary habits are all going up with global food consumption. Rice (*Oryza sativa* L.) is one of the most significant cereal crops in the world, growing in a wide range of climate regions (Abd El-Hakim et al., 2022; Qi et al., 2022). Rice is one of the major cereal crops globally and is utilized by approximately half of the world's population (Shaheen et al., 2022). In Egypt, where the area allocated to rice farming is ~555,147 ha, and the average yield is ~8.8 t/ha. Comparatively, the world cultivated rice area is ~167.13 million ha with 4.7 t/ha as yield average (Khan et al., 2021; Soto-Gómez and Pérez-Rodríguez, 2022).

Foliar application of Zn resulted in increasing rice grain yield in comparison with the control treatment (Fageria and Baligar, 2005). It has been observed that applying Zn at 45 days after planting has the most correlation with the yield of rice (Slaton et al., 2005). In rice, foliar spraying of Zn is a particularly advantageous method to increase yield, quality, and grain Zn concentration compared with soil application, as it avoids the complex soil interactions that limit Zn uptake through a plant's roots (Mabesa et al., 2013). Foliar application of Zn represents an effective practice to enhance grain Zn significantly. In wheat, spraying of Zn fertilizer increased both productivity and grain Zn concentration up to 3- or 4-folds as a foliar spray (Cakmak, 2008). Also, Phattarakul et al. (2012) indicated that foliar spray of Zn to rice grown under field conditions caused a greater enhanced yield and grain Zn content. Application of Zn fertilizers improved dry matter, grain yield, and grain Zn concentration in rice (Fageria et al., 2011). In rice, foliar spray of Zn significantly enhanced grain yield, quality, and Zn accumulation in the grain (Yuan et al., 2013). Foliar Zn spray was significantly affected in all measured characteristics of rice such that the highest values of grain yield and yield components of rice were recorded when Zn was applied at the booting stage (Guo et al., 2016).

Moreover, the application of micronutrients enhanced the content of Zn and the other micronutrients in rice grains and straw yield (Bana et al., 2022; Haefele et al., 2022). A high level of antioxidants was found in the dehusked grains compared with milled rice after the application of micronutrients. The application of micronutrients increased rice yield by 18% by increasing physiological characteristics, leading to high grain yield, harvest index (HI), and quality of grains (Panhwar et al., 2015). Usage of Zn increased protein and iron (Fe) concentrations in grain and straw yields of rice and improved the grain yield (Rehim et al., 2014; Abdelsalam et al., 2018), and it also enhanced the yield and yield components and nutrient accumulation in rice plants. Additionally, it significantly enhanced grain yield with respect to control. The foliar application of Zn had a better effect than the soil form for some parameters in rice grain (Kheyri et al., 2019). Positive effects on the productivity of rice cultivated in saline-sodic soils can be observed from foliar application of fertilizer compounds that contain essential micronutrients, such as Mn, Fe, and Zn, resulting in proper growth in saline and alkaline soil conditions and improving tolerance to stress. The presence of Zn in commercial compound microfertilizers is essential for the improvement of rice growth and yield (Kheir et al., 2019; Fouda et al., 2020; Kandil et al., 2020a; Sukyankij et al., 2020). Finally, the foliar applications of Zn provide nutrients to plants, revive the soil to an organic state, enhance plant growth, and reduce environmental pollution compared to soil-applied fertilizers (Sabir et al., 2014).

Mostly, all crops, as well as calcareous, sandy soils, and soils high in phosphorus and silicon, are likely to be Zn-deficient, particularly in salinity-affected soils. The aim of this study was to see how different Zn concentrations (1,500, 2,000, and 2,500 mg/L) as a foliar spray (after 30 and 45 days from rice transplanting) affected agronomic, yield, and kernel quality traits in some Egyptian and Iraqi rice (*O. sativa* L.) cultivars, as well as to confirm the genotoxic effect of Zn concentrations on pollen grain viability and aberrations to find the same dose that did not cause any.

## Materials and Methods

### Experimental Site Description

The field experiments were conducted at El-Behira Governorate, Egypt in both seasons 2018 and 2019. The soil physical and chemical analyses were performed (Table 1) as recommended by Chapman et al. (2001). In both seasons, the preceding crop was maize.

**Table 1:** Physical and chemical properties of the experimental soil site in both seasons (2018 and 2019), in addition to the composition of zinc commercial product (ZnO).

**Soil properties**	**Growing season**	
	**2018**	**2019**
**A- mechanical analysis**		
Sand	11.50	11.70
Silt	42.10	42.10
Clay	46.40	46.20
Soil texture	Clay loam	Clay loam
**B- Chemical analysis**		
pH (1:1)	8.10	8.00
E.C. (1:1) dS/m	4.10	4.00
Soluble cations (1:2)		
K ^+^	1.50	1.40
Ca ^++^	11.20	11.40
Mg ^++^	8.30	8.50
Na ^+^	9.60	10.70
**Soluble anions (1:2)**		
CO ^−3^ + HCO ^−3^	2.60	2.90
CL ^−^	16.60	18.10
SO ^−4^	11.40	11.50
Calcium carbonate (%)	6.50	6.60
Nitrogen (%)	1.30	1.25
Available P (mg/kg)	2.70	2.60
Available Zn (mg/kg)	0.55	0.45
Organic matter (%)	1.88	1.90
**Composition of zinc commercial product**	Super ZnO -fertilizer (%)	
Zinc (ZnO)	10	
Total Vitamins	1	
Total Seaweed extracts	2	
Total Amino acids	0	
Total Organic acids	25	

### Experiment Layout and Design

Every experimental was a split plot design with 3 replicates, 5 rice cultivars (two Egyptian cultivars, Sakha 101, and Giza 178; and three Iraqi cultivars, Yasmeen, Fourate, and Amber 33), randomly distributed in the main plots. The sub-plots were allocated by four foliar applications of Zn concentrations (water spray, 1,500, 2,000, and 2,500 mg/L). Foliar spray application of Zn was performed two times (30 and 45 days after rice transplanting: tillering and panicle initiation stages) on rice plants. The composition of the structured fertilizer Zn was measured. Each sub-plot measured 10.50 m^2^ (3 m long × 3.5 m wide). The grains of the Egyptian and Iraqi rice cultivars were obtained from rice, from the research sections of the Agriculture Research Center (ARC), Ministry of Agriculture and Land Reclamation (MALR) in Egypt and Iraq, respectively. The seeding rate was (120 kg) seeds/ha, which were sown in the nursery (1/12 from the total area of the permanent field). The seedlings (after 30 days from sowing in the nursery) were handily transplanted in the permanent field on the 12th and 15th of May in 2018 and 2019, respectively, in the first and the second seasons, spacing (20 × 20 cm) at 3 seedlings/hill, and the 1st irrigation was utilized at 25 days after sowing, and the plants were irrigated every 6 days until the dough growth stage.

Mineral *N* fertilizer (168 kg N/ha) was added in two doses. The 1st dose (1/3 of the total N/ha dose) was added at the 1st irrigation (after 25 days of sowing), and the 2nd dose (2/3 of the total N/ha) was added at the second irrigation (25 days after the first dose). N fertilizer (46.5% N) was added as urea. Superphosphate fertilizer was applied before sowing at 60 kg P_2_O_5_/ha. Potassium (K) fertilizer was applied before sowing (during seedbed formulation) at 60 kg K_2_O/ha in the form of potassium sulfate (48% K_2_O). All the other cultural practices were followed as the recommendation of the Ministry of Agriculture and Land Reclamation recommendations.

### Physical Characterization of the Zn Fertilizer

Aqueous dispersions of the treatment concentrations were diluted 20 times in distilled water at ambient temperature before analysis and then transferred to disposable cuvettes with a detection angle of 173° at room temperature. The particles were evaluated by dynamic light scattering (DLS) on a Malvern Zetasizer Series, and the hydrodynamic size was represented with Z-average values in meters. The zeta potential (ζ-potential) measurements were carried out in disposable folded capillary cells, and the electrophoretic motilities were converted to ζ-potential using the Smoluchowski model with Henry's function value of 1.50. All determinations were reported as the average of three measurements.

### The Studied Characters

#### Yield and Yield Components' Characters

At harvest, plant height (cm) and panicle length (cm) were mustered from 20 plants; grain yield (t/ha), straw yield (t/ha), and biological yield (t/ha) were mustered from one square meter; harvest index (HI%) = grain yield (t/ha)/ biological yield (t/ha) ^*^ 100- and 1,000-grain weight (g) was measured an average from three samples during both seasons. The protein % in grains were measured by assessing the total nitrogen in the grain multiplied by 5.75 to obtain the protein %, according to a study by Chemists (1990). The grain Zn content (mg/kg) was established by the Vanadomolybdate yellow technique described by Jackson (1958), and the amount of color developed was read on a spectrophotometer at 460 nm.

#### Kernel Quality Traits

To estimate the grain kernel quality traits, 150 g of each rice sample was taken at random; the rice samples were cleaned and dehulled with an experimental Satake huller machine, polished in a Satake miller, and estimated according to the standard evaluation system (Aamer and Tabl, 2019). The dimensions of the rice grains (length, thickness, and width) were measured using a Satake shep tester (Model MK-200, Japan) with a range of 0–20 mm and an accuracy of 0.01 mm (Qureshi et al., 2019). Grain hardness was measured using a grain hardness tester; ten grains were tested from each rice sample (Islam et al., 2004). Gel consistency was revealed using the procedure described by Cagampang et al. (1973). The gelatinization temperature was recorded according to the study by Little (1958). The elongation ratio was assessed according to the technique described by Azeez and Shafi (1966); Cruz and Khush (2000).

The amylose content was assessed using the basic procedure reported by Juliano (1971); the paddy rice was mechanically cleaned, and impurities were removed based on shape, size, and specific weight. The cleaned rice grains were placed in a pressure tank and soaked for −150 min in water kept in the circulation at 65, 70, and 75°C. When the rice reached the water temperature, the water supply was shut off, and hydrostatic pressure of 4–6 kg/cm was used by admitting compressed air. The 2nd heating or cooking period began by reducing and readmitting water heated to a very high temperature to make sure complete gelatinization of the starches. The water is then drained away, and the rice grains with a moisture content of ~30–35% (Kimura et al., 1995) were dried to 13% at room temperature (25°C).

#### Cytological Characters

**Figure 1:**
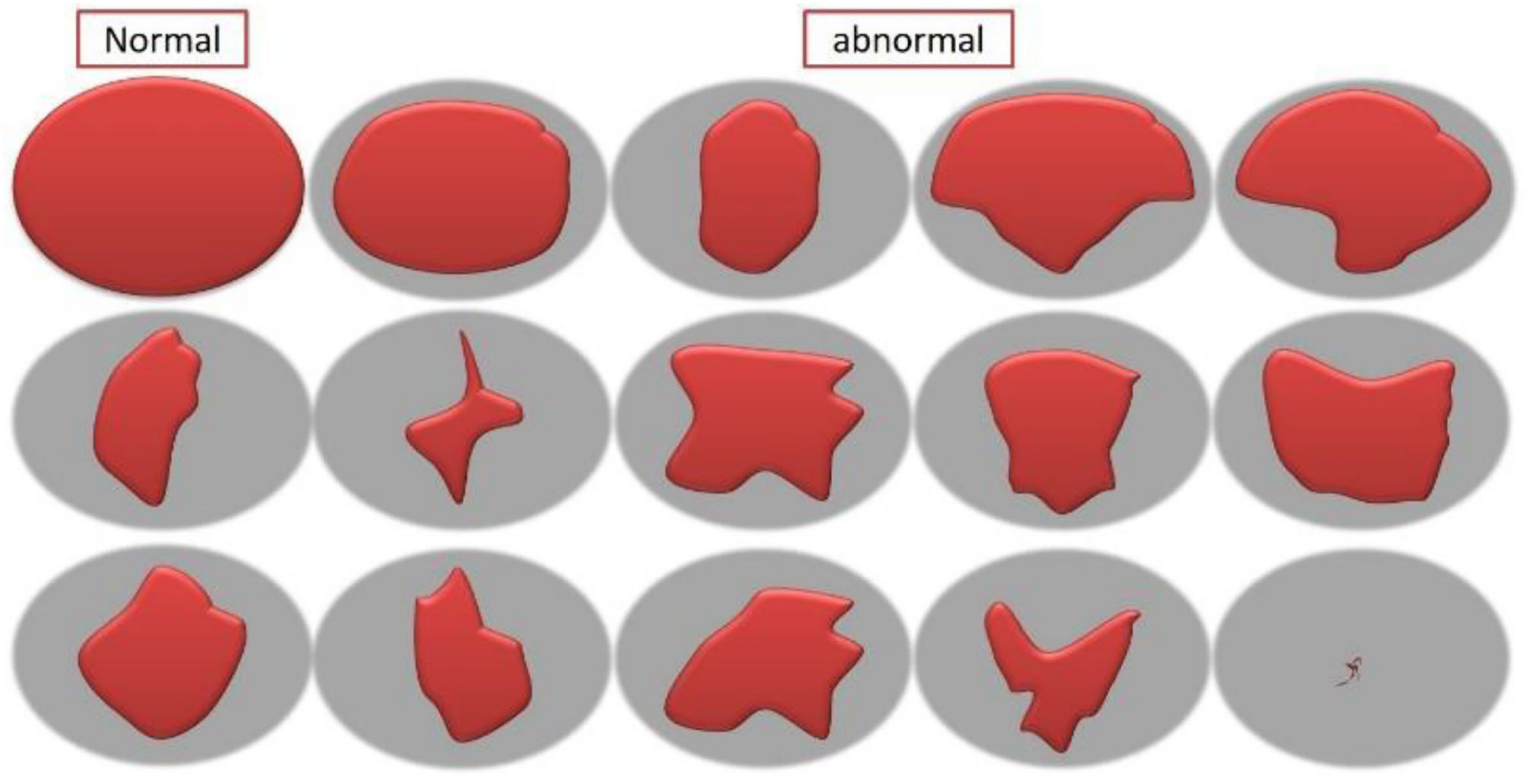
Stylized forms of pollen grain shape aberrations observed in rice affected by Zn fertilizer.

***Assessment of Pollen Fertility and Sterility***. Between 9 and 11 a.m., the anthers from fresh, mature 5-mm buds were chosen from the area after several Zn fertilizer treatments to examine the pollen grain sterility. The viable pollen grains that were fully turgid, partially turgid, fully dark, dark blue, black-stained nuclei, multinucleate, acetocarmine stained, or non-stained were calculated under a microscope (stylized forms of pollen grain shape aberrations found in Figure 1), and the pollen aberrations percentage and pollen sterility were calculated matching to Haroun (1995) and Mohanty et al. (2004) using the following formula:


$$\begin{array}{l} Pollen\;sterility\;\%\\ =\frac{Number\;of\;sterile\;pollen\;in\;microscopic\;field}{Total\;number\;of\;pollen\;in\;microscopic\;field}\;*100\\ Aberrations\;\%\\ =\frac{Number\;of\;abnormal\;pollen\;in\;microscopic\;field}{Total\;number\;of\;pollen\;in\;microscopic\;field}\;*100 \end{array}$$

To assess the pollen viability, the acetocarmine coloration of the pollen grains was used. Pollen of the normal size was equally stained with acetocarmine that was considered fertile pollen, while those partially stained appeared as shrunken pollen. Almost one thousand pollen grains were examined and evaluated according to the methods of Mosa et al. (2021). About 1 drop of acetocarmine was moved on to the slides, and the pollen of fresh mature bud was scattered on the solution and covered with a coverslip to identify the viability of pollen (Mosa et al., 2021). The stylized images of variations and aberrations in the forms of the pollen grains are drawled and detected.

### Statistical Analysis

The results obtained were exposed to an analysis of variances defined by Gomez and Gomez (1984). The treatment means were evaluated using the least significant difference (LSD) test at a level of 5.0% probability. All statistical analyses were done by the CoStat computer software package 39 CoStat (2005).

## Results and Discussion

This study aimed to evaluate the Zn foliar fertilizer for Egyptian and Iraqi rice in terms of yield, quality, and genotoxicity. Before applying this fertilizer to rice, it should be fully characterized to outline its efficiency based on its structure. Below are the physical characteristics of the commercial fertilizer examined using dynamic light scattering (DLS) and scanning electron microscopy (SEM).

### Microparticle Characterization

**Figure 2:**
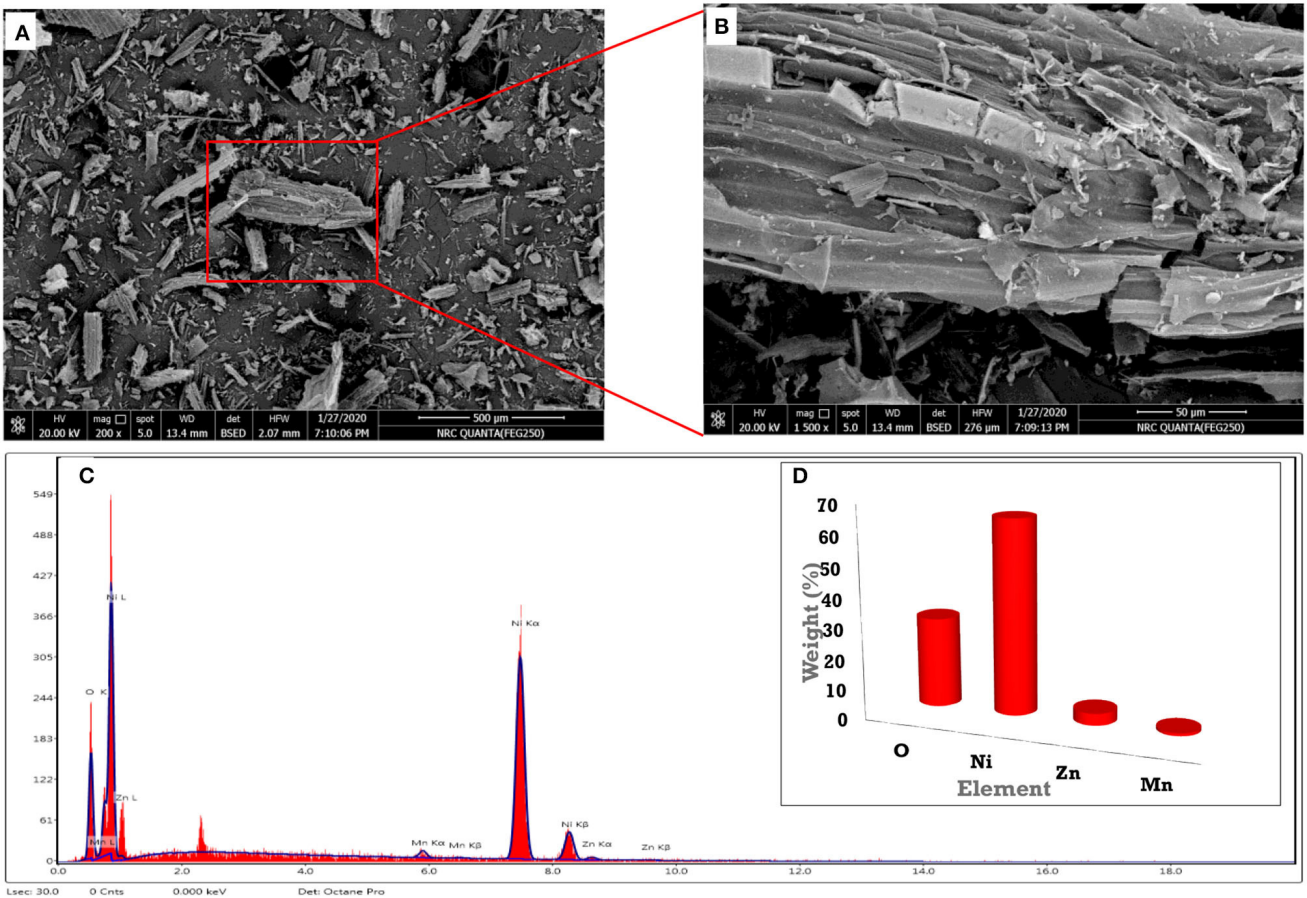
**(A,B)** SEM at low and high magnifications and **(C,D)** EDX of the commercial Zn fertilizer.

**Figure 3:**
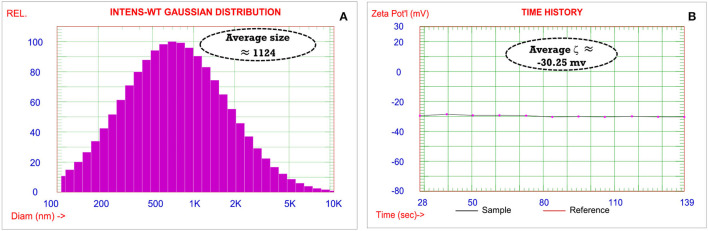
**(A)** analysis of particle size **(B)** zeta potential of the commercial Zn fertilizer.

The microcrystalline structure of the commercial material was examined by SEM-EDX before application. Commercial Zinc was subjected to centrifugation at 10,000 rpm for 2 h, and the obtained powder was freeze-dried for 24 h. SEM images were taken by various magnifications of 200 and 1,500 as demonstrated in Figures 2A,B, respectively. It is observed that the microstructure particles had regular shapes. Additionally, the particles formed had a rough surface and were >1 mm due to agglomeration and the deposition of layers. Furthermore, EDX was performed to identify the elemental composition (Figure 2C). The scanned material had many elements with different weight percentages, as displayed in (Figure 2D). From EDX (Figure 2C), it is revealed that the material has a high percentage of Ni in a high percentage, followed by O, Zn, and Mn. The increase of Ni % due to the commercial zinc structure. The absence of the C revealed that fertilizer is inorganic, not an organic compound. From the EDX, it can be concluded that the material is an inorganic compound with many elements in varying ratios. “The hydrodynamic diameter, polydispersity index (PDI), and zeta potential” were evaluated by “DLS” using the “Zetasizer.” All tests were executed in triplicates at 25°C. Before testing, samples were degassed for 5 min using a “Tabletop Ultrasound Cleaner 2510” “(Branson Ultrasonic, Danbury, CT, USA).” As represented in Figure 3A, the average hydrodynamic size is ~1 μm with a polydispersity index (PDI) of 0.9. The particle size analysis data confirmed that the compound has heterogeneous size particles with a polydispersity index of >0.5. The zeta potential was ~30 mv (Figure 3B). This highly negative surface charge is due to the electrostatic repulsion of particles in suspension, and the effective electric charge on the particle surface increases the electric force between them. Therefore, the stability of the particles increases, affirming the stabilization of the microstructure feature of the material. It can be assumed that the positive charge of the microparticles is directly correlated with the pH value of the colloidal solution.

### Effect of Zn Fertilizer on Yield and Yield Components

The obtained results in Tables 2, 3 revealed the effect of foliar application of Zn on plant attributes of rice cultivars and their interaction in 2018 and 2019. There were significant differences among the five rice cultivars regarding plant height, grain, straw, biological yield, harvest index (H%), 1,000-grain weight (g), panicle length, protein percentage (%), and grain Zn content (mg/kg). Iraqi rice cultivar Amber 33 showed the tallest plants, 113.56 and 120.98 cm, during both seasons, respectively, compared with the other cultivars. On the other hand, the Egyptian rice cultivar Giza 178 showed the highest mean values of grain yield (8.73 and 9.08 t/ha), straw yield (9.97 and 10.51 t/ha), and biological yield (18.70 and 19.59 t/ha), respectively, in both seasons followed by Skha101 which displayed the maximum HI (46.21%) in the first season, but Giza 178 showed the highest HI (46.50%), respectively, in both seasons as shown in Table 2. However, in Table 3, Sakha 101 displayed the maximum mean values of 1,000-grain weight (21.48 and 21.07 g), respectively, in both seasons and the longest panicle length (11.42 cm) in 2018, while Giza 178 showed the longest panicle (11.96 cm) in 2019; however, Giza 178 recorded the highest content of protein % (9.00), and Yasmeen cultivar gave the highest % of the protein in grain (9.00%) and the highest content of Zn in grain (44.15 and 44.80 ppm) in both seasons. There was an insignificant variation between the two Egyptian cultivars Giza 178 and Sakha 101, in either season. Meanwhile, Fourate and Amber 33 showed the lowest yields in the 2018 and 2019 seasons. Plant characteristics depend on the genetic attributes of a plant and environmental requirements. The rice varieties and growth conditions have the largest effect on the agronomic effectiveness of foliar Zn spray to improve grain yields, its components, and Zn contents (Phattarakul et al., 2012; Boonchuay et al., 2013; Mabesa et al., 2013). These results are in close agreement with the study of Sadimantara and Cahyono (2014), and Ghasemi et al. (2017) who reported that the difference among rice cultivars is due to genetic differences.

**Table 2:** Plant attributes of some rice cultivars affected by the concentration of Zn during the 2018 and 2019 seasons.

**Attributes**		**Season 2018**					**Season 2019**				
	**(A) Cultivars**	**(B) Foliar application of Zn (mg/L water)**				**Mean (A)**	**(B) Foliar application of Zn (mg/L water)**				**Mean (A)**
		**Water**	**1,500**	**2,000**	**2,500**		**water**	**1,500**	**2,000**	**2,500**	
**Plant height (cm)**	Sakha 101	74.70	75.03	68.03	76.80	73.64	82.70	83.03	76.03	85.80	81.89
	Giza 178	76.13	79.70	79.37	87.37	80.64	82.13	87.70	87.37	95.37	88.14
	Yasmeen	79.00	85.70	91.23	100.57	89.13	79.67	92.70	97.80	109.57	94.94
	Fourate	72.47	70.13	88.03	85.47	79.03	78.57	75.13	96.03	91.23	85.24
	Amber 33	85.90	114.67	126.33	127.33	113.56	91.57	122.67	134.33	135.33	120.98
Mean (B)		77.64	85.05	90.60	95.51		82.93	92.25	98.31	103.46	
LSD at 0.05 (A)			4.65						4.46		
LSD at 0.05 (B)			3.93						3.34		
LSD at 0.05 (AB)			8.78						7.47		
**Grain yield (t/ha)**	Sakha 101	6.97	8.03	9.13	9.30	8.36	6.47	8.53	9.63	9.40	8.51
	Giza 178	7.90	8.47	8.90	9.63	8.73	7.13	8.97	9.40	10.80	9.08
	Yasmeen	6.63	7.80	8.97	8.43	7.96	6.13	8.30	9.47	8.93	8.21
	Fourate	5.97	7.57	7.90	8.23	7.42	6.67	8.07	8.57	8.67	8.00
	Amber 33	6.83	7.80	7.97	7.57	7.54	6.07	8.30	8.47	8.00	7.71
	Mean (B)	6.86	7.93	8.57	8.63		6.49	8.43	9.11	9.16	
LSD at 0.05 (A)			0.68						0.67		
LSD at 0.05 (B)			0.60						0.54		
LSD at 0.05 (AB)			1.35						1.20		
**Straw yield (t/ha)**	Sakha 101	7.33	9.63	10.20	10.27	9.36	8.13	9.93	11.20	10.90	10.04
	Giza 178	8.73	9.97	10.33	10.85	9.97	8.60	10.63	10.90	11.90	10.51
	Yasmeen	7.47	9.30	9.90	9.60	9.07	7.83	10.00	11.03	10.50	9.84
	Fourate	7.00	9.07	9.50	9.73	8.83	7.47	9.77	10.13	10.47	9.46
	Amber 33	7.50	9.23	9.47	9.40	8.90	8.13	9.87	9.87	9.57	9.36
	Mean (B)	7.61	9.44	9.88	9.97		8.03	10.04	10.63	10.67	
LSD at 0.05 (A)			0.72						0.53		
LSD at 0.05 (B)			0.50						0.49		
LSD at 0.05 (AB)			1.21						1.10		
**Biological yield (t/ha)**	Sakha 101	14.30	17.66	19.33	19.57	17.72	14.60	18.46	20.83	20.30	18.55
	Giza 178	16.63	18.44	19.23	20.48	18.70	15.73	19.60	20.30	22.70	19.58
	Yasmeen	14.10	17.10	18.87	18.03	17.03	13.96	18.30	20.50	19.43	18.05
	Fourate	12.97	16.64	17.40	17.96	16.24	14.14	17.84	18.70	19.14	17.46
	Amber 33	14.33	17.03	17.44	16.97	16.44	14.20	18.17	18.34	17.57	17.07
	Mean	14.47	17.37	18.45	18.60		14.53	18.47	19.73	19.83	
LSD at 0.05 (A)			1.34						1.17		
LSD at 0.05 (B)			1.10						0.99		
LSD at 0.05 (AB)			2.46						2.21		
**Harvest index (HI %)**	Sakha 101	48.74	45.47	47.23	47.52	47.24	46.21	46.23	46.31	45.88	46.16
	Giza 178	47.50	45.93	46.28	47.02	46.69	45.77	46.31	47.58	46.37	46.50
	Yasmeen	47.02	45.61	47.54	46.76	46.73	45.36	46.20	45.96	45.49	45.75
	Fourate	46.03	45.49	45.40	45.82	45.69	45.24	45.83	45.30	45.83	45.55
	Amber 33	47.66	45.80	45.70	44.61	45.94	45.68	46.18	45.53	45.17	45.64
Mean (B)		47.39	45.66	46.43	46.35		45.65	46.15	46.13	45.75	
LSD at 0.05 (A)			1.55						1.09		
LSD at 0.05 (B)			1.22						0.98		
LSD at 0.05 (AB)			2.74						2.20		

**Table 3:** Plant attributes of some rice cultivars affected by the concentration of Zn during the 2018 and 2019 seasons.

**Attributes**		**Season 2018**					**Season 2019**				
	**(A) Cultivars**	**(B) Foliar application of Zn (mg/L water)**				**Mean (A)**	**(B) Foliar application of Zn (mg/L water)**				**Mean (A)**
		**Water**	**1,500**	**2,000**	**2,500**		**Water**	**1,500**	**2,000**	**2,500**	
**1,000- grain weight (g)**	Sakha 101	18.73	21.13	23.30	22.77	21.48	18.57	20.63	22.80	22.27	21.07
	Giza 178	19.07	20.73	22.70	23.03	21.38	18.97	20.23	22.20	22.53	20.98
	Yasmeen	14.07	14.73	16.70	17.03	15.63	13.83	15.07	16.30	16.53	15.43
	Fourate	18.00	19.93	20.27	21.20	19.85	17.97	19.43	19.77	20.70	19.47
	Amber 33	18.00	19.80	20.57	18.73	19.28	17.33	19.30	20.50	18.83	18.99
Mean (B)		17.57	19.26	20.71	20.55		17.33	18.93	20.31	20.17	
LSD at 0.05 (A)			1.14						0.96		
LSD at 0.05 (B)			0.80						0.79		
LSD at 0.05 (AB)			1.79						1.77		
**Panicle length (cm)**	Sakha 101	8.67	13.00	11.00	13.00	11.42	9.33	12.00	12.00	13.33	11.67
	Giza 178	9.00	11.00	11.33	13.67	11.25	9.50	12.00	12.33	14.00	11.96
	Yasmeen	7.00	9.63	9.33	11.67	9.41	9.00	10.87	11.33	12.00	10.80
	Fourate	8.67	12.00	11.00	13.00	11.17	8.67	12.00	12.00	13.67	11.59
	Amber 33	8.27	12.33	13.00	11.00	11.15	8.60	12.67	13.67	11.33	11.57
	Mean (B)	8.32	11.59	11.13	12.47		9.02	11.91	12.27	12.87	
LSD at 0.05 (A)			0.53						0.99		
LSD at 0.05 (B)			0.59						0.71		
LSD at 0.05 (AB)			1.31						1.59		
**Grain protein (%)**	Sakha 101	6.77	8.91	9.45	9.40	8.63	7.33	9.21	9.59	9.58	8.93
	Giza 178	7.32	9.18	9.32	10.18	9.00	7.62	8.67	9.33	10.23	8.96
	Yasmeen	7.27	9.25	9.67	9.63	8.96	7.28	9.80	9.57	9.33	9.00
	Fourate	8.05	8.64	8.70	9.52	8.73	7.90	9.43	9.09	9.00	8.86
	Amber 33	7.20	8.20	8.48	8.90	8.20	7.33	7.87	8.33	9.40	8.23
	Mean (B)	7.32	8.84	9.12	9.53		7.49	9.00	9.18	9.51	
LSD at 0.05 (A)			0.36						0.56		
LSD at 0.05 (B)			0.40						0.59		
LSD at 0.05 (AB)			0.91						1.33		
**Grain Zn content**	Sakha 101	40.50	43.72	44.52	47.87	44.15	40.67	44.52	45.32	48.70	44.80
	Giza 178	38.83	40.58	42.50	45.83	41.94	38.43	41.91	43.92	46.78	42.76
	Yasmeen	34.83	36.58	38.50	41.83	37.94	35.43	38.91	40.92	43.78	39.76
	Fourate	39.80	46.13	44.67	42.33	43.23	36.33	46.95	44.94	41.70	42.48
	Amber 33	39.50	44.10	44.57	42.88	42.76	38.93	36.29	43.11	43.61	40.49
	Mean	38.69	42.22	42.95	44.15		37.96	41.72	43.64	44.91	
LSD at 0.05 (A)			2.27						2.28		
LSD at 0.05 (B)			1.57						2.15		
LSD at 0.05 (AB)			3.52						4.82		

The results shown in Tables 2, 3 revealed that there was a significant effect of Zn on all plant attributes. Increasing fertilizer levels up to 2,500 mg/L increased all the studied characteristics. Specifically, Zn fertilizer at the rate of 2,500 mg/L achieved the maximum mean value of plant height (95.51 and 103.46 cm), grain yield (8.63 and 9.16 t/ha), straw yield (9.97 and 10.67 t/ha), biological yield (18.60 and 19.83 t/ha), panicle length (12.47 and 12.87 cm), protein (9.53 and 9.51%), and Zn content (44.15 and 44.91 ppm), followed by foliar application of 2 g/L of Zn that showed the highest values of HI (46.66 and 46.15%) and 1,000-grain weight (20.71 and 20.31), respectively, whereas the control treatments (water spray) showed the lowest values in both seasons. Current results are confirmed by Ghasemi et al. (2017), who stated the increased yield and its components.

Also, Tables 2, 3 show the significant interaction between rice cultivars and foliar application of Zn, whereas fertilizing Giza 178 with a foliar application at the rate of 2,500 mg/L achieved the highest mean values of grain yield (9.63 and 10.80 t/ha), straw yield (10.85 and 11.90 t/ha), biological yield (20.48 and 22.70 t/ha), harvest index (47.14 and 47.58%), 1,000-grain weight (23.03 and 22.53 g), panicle length (13.67 and 14.00 cm), protein (10.18 and 10.23%), and Zn content (45.83 and 46.78 ppm) in the two seasons, respectively, followed by the rate of 2,000 mg/L of Zn. On the other hand, Amber 33 with Zn application at the rate of 2,500 mg/L gave the longest rice plants (127.33 and 135.33 cm) in both seasons, respectively.

Specifically, Zn fertilizer at the rate of 2,500 mg/L achieved the maximum mean value of plant height (95.51 and 103.46 cm), grain yield (8.63 and 9.16 t/ha), straw yield (9.97 and 10.67 t/ha), biological yield (18.60 and 19.83 t/ha), panicle length (12.47 and 12.87 cm), protein (9.53 and 9.51%), and Zn content (44.15 and 44.91 ppm); followed by foliar application of 2 g/L of Zn that showed the highest values of HI (46.66 and 46.15%) and 1,000-grain weight (20.71 and 20.31), respectively, followed by foliar application of Zn at the rate of 2,000 mg/L, whereas the control treatments (water spray) showed the lowest values in both seasons.

**Figure 4:**
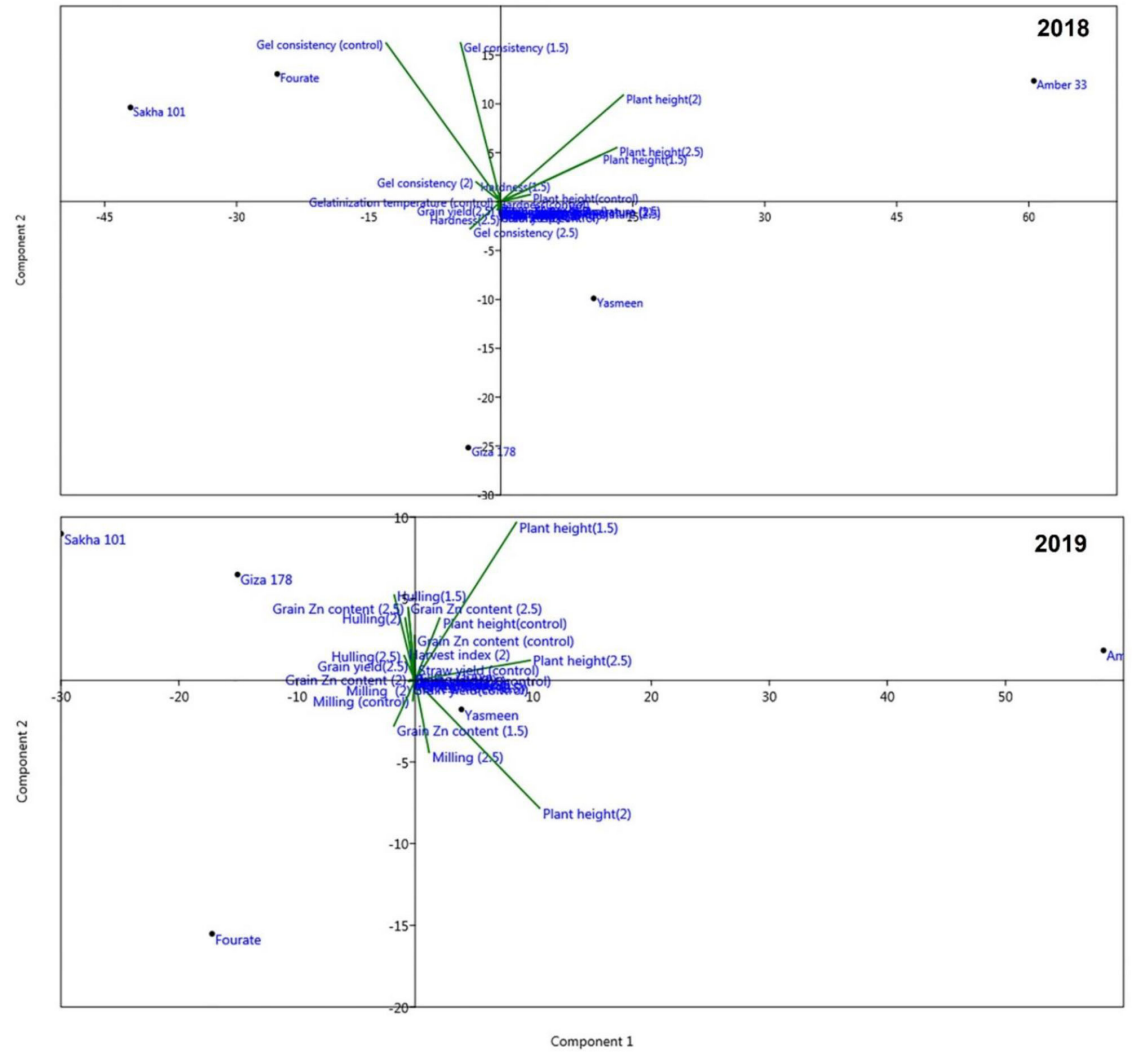
Biplot perceptual mapping shows the genetic similarity distances between five rice genotypes treated with different concentrations of Zn based on physiological and kernel quality attributes in 2018 and 2019 according to Euclidean distance and the unweighted pair group method with arithmetic mean (UPGMA) algorithm.

Biplot perceptual mapping to study the interaction between different rice genotypes, physiological traits, and kernel quality traits in the 2018 season are illustrated in Figure 4. The genotypes were clustered into two groups; the first group included Sakha 101 and Fourate genotypes and the second group included other three genotypes (Giza 178, Amber 33, and Yasmeen). Biplot indicated that plant height from plant attributes and gel consistency from kernel quality traits are the most significant characteristics, whereas the arrow length refers to the most powerful trait and the direction of the arrow points to the center referred to the significant plant traits. Data of plant attributes and kernel quality traits are used to compute biplot perceptual mapping for the coordination and interaction of genotypes, and the physio-kernel quality traits in 2019 are illustrated in Figure 4. The data from the biplot clarified that the genotypes were clustered into two groups: one group contained Sakha 101 and Fourate genotypes and the other group contained Giza 178, Amber 33, and Yasmeen genotypes. The main significant factor was plant height followed by milling % and grain Zn content.

**Figure 5:**
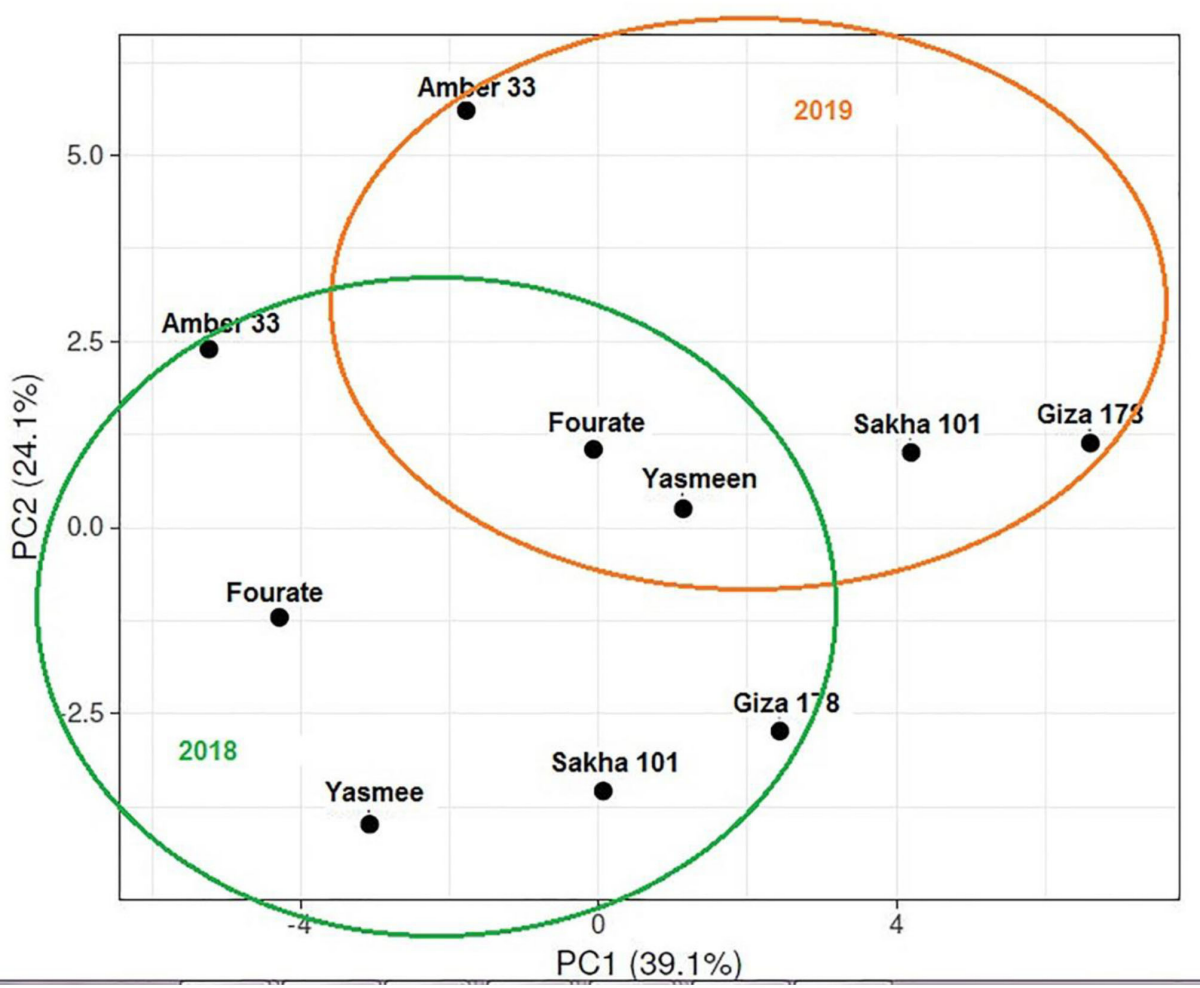
Principle component analysis (PCA) for all physiological traits and kernel quality traits of five rice genotypes under Zn treatments for two seasons (2018 and 2019).

Principle component analysis (PCA) for all physio-kernel quality traits of the five rice genotypes under Zn treatments is illustrated in Figure 5. PCA explained the maximum variation interaction by morphological and physiological traits which concluded that the studied genotypes were coordinated into two groups in two seasons and the groups were interposed. PCA is a multivariate analysis for data used in the visualization of relationships, similarities, and dissimilarities among various plant parameters against different treatments. The first two principal components (PC1 and PC2) with a total variance of 39.1 and 24.1%, respectively. These values are considered the best measures for the quality of coordination and the strength of the genotypes–morpho-physiological relationship.

### Kernel Quality Traits

The results in Tables 4, 5 showed the effect of foliar application of Zn on the quality of the rice kernel cultivars and their interaction during both seasons. The rice kernel quality was significantly differed in hulling (%), broken (%), hardness, grain length, shape, amylose (%), gel consistency, and gelatinization temperature. The results indicated that Giza 178 and Skha101 showed the highest values for most kernel quality traits, such as hulling (%), grain shape, gel consistency (GC), and gelatinization temperature (GT), as compared to the other rice kernel in both seasons. Meanwhile, the studied kernel characters in rice cultivars did not differ significantly in the following parameters, i.e., milling (%), hardness (%), and grain thickness. Also, the results revealed that Zn treatment significantly increased most of the positive effects on kernel quality traits, i.e., hulling (%), milling (%), hardness (%), grain thickness, grain shape, amylose content, GC, and GT in the two seasons. The results showed no significant increase in grain length in the control treatment (water spray), and it showed the smallest values in both harvest seasons. These findings are in accord with those reported by Ruangthep (2018), which indicated that the Zn element increased the rice quality. The interaction between rice cultivars was significant as shown in Tables 3, 4, where fertilizing Giza168 with the rate of 2.5 g /L of Zn recorded the highest values of hulling%, broken%, and hardness% whereas on the other hand, Sakha101 + 2.5 g /L of Zn achieved the highest values of GC, GT rice cultivar Yasmeen with 2.5 g/L of Zn gave the highest values of grain shape, and amylose % and cv. Fourate + 2,500 mg/L of Zn gave the highest grain length and thickness during the two seasons.

**Table 4:** Kernel quality traits of five rice cultivars affected by the concentration of Zn during the 2018 and 2019 seasons.

**Attributes**		**Season 2018**					**Season 2019**				
	**(A) Cultivars**	**(B) Foliar application of Zn (mg/L water)**				**Mean (A)**	**(B) Foliar application of Zn (mg/L water)**				**Mean (A)**
		**Water**	**1,500**	**2,000**	**2.500**		**water**	**1,500**	**2,000**	**2,500**	
**Hulling (%)**	Sakha 101	71.73	78.17	77.93	80.63	77.12	69.67	79.43	79.33	81.50	77.48
	Giza 178	68.67	78.00	79.90	82.00	77.14	67.00	79.12	80.52	82.78	77.36
	Yasmeen	69.67	72.00	74.47	78.93	73.77	68.67	71.85	75.03	79.28	73.71
	Fourate	69.00	71.13	74.40	80.00	73.63	68.90	71.70	74.45	79.67	73.68
	Amber 33	67.33	68.47	73.73	78.33	71.97	68.12	69.03	74.45	77.00	72.15
Mean (B)		69.28	73.55	76.09	79.98		68.47	74.23	76.76	80.05	
LSD at 0.05 (A)			1.50						1.60		
LSD at 0.05 (B)			2.23						2.16		
LSD at 0.05 (AB)			4.99						4.82		
**Milling (%)**	Sakha 101	69.00	70.40	70.67	70.47	70.14	67.60	70.80	71.07	70.87	70.09
	Giza 178	68.00	70.70	72.00	70.77	70.37	67.93	70.63	72.40	71.17	70.53
	Yasmeen	68.67	69.17	68.07	75.47	70.35	68.60	69.57	68.47	75.40	70.51
	Fourate	68.37	69.37	71.40	77.33	71.62	69.30	69.77	71.80	77.47	72.09
	Amber 33	66.37	69.27	68.83	77.67	70.54	67.17	69.20	69.23	78.27	70.97
	Mean (B)	68.08	69.78	70.19	74.34		68.12	69.99	70.59	74.64	
LSD at 0.05 (A)			Ns						ns		
LSD at 0.05 (B)			1.62						2.41		
LSD at 0.05 (AB)			3.63						3.54		
**Broken (%)**	Sakha 101	5.90	5.87	6.47	4.77	5.75	6.05	6.12	6.97	4.97	6.03
	Giza 178	5.90	6.10	5.17	5.47	5.66	6.08	6.35	5.42	5.72	5.89
	Yasmeen	3.93	5.07	5.70	4.70	4.85	4.10	5.32	5.95	4.60	4.99
	Fourate	4.73	4.07	4.23	4.97	4.50	4.98	4.32	4.48	5.03	4.70
	Amber 33	4.50	5.67	4.73	4.33	4.81	4.50	5.50	4.98	5.53	5.13
	Mean (B)	4.99	5.36	5.26	4.85		5.14	5.52	5.56	5.17	
LSD at 0.05 (A)			0.55						0.59		
LSD at 0.05 (B)			Ns						ns		
LSD at 0.05 (AB)			1.35						1.31		
**Hardness (%)**	Sakha 101	7.07	7.93	7.17	6.50	7.17	6.60	7.40	6.82	6.47	6.82
	Giza 178	5.40	6.23	8.17	8.13	6.98	5.05	5.97	7.81	7.78	6.65
	Yasmeen	5.53	5.77	6.23	7.80	6.33	5.43	5.42	5.88	7.45	6.05
	Fourate	5.87	6.63	5.97	7.33	6.45	5.73	6.28	5.73	7.65	6.35
	Amber 33	5.70	7.07	6.73	6.40	6.48	5.42	6.72	6.50	6.65	6.32
	Mean	5.91	6.73	6.85	7.23		5.65	6.36	6.55	7.20	
LSD at 0.05 (A)			Ns						ns		
LSD at 0.05 (B)			0.65						0.63		
LSD at 0.05 (AB)			1.45						1.42		
**Grain length (mm)**	Sakha 101	5.14	5.52	5.77	5.57	5.50	5.20	5.58	5.83	5.63	5.56
	Giza 178	6.10	5.89	5.61	6.03	5.91	6.16	5.94	5.67	6.09	5.97
	Yasmeen	5.49	6.11	6.20	5.82	5.91	5.73	6.17	6.26	5.88	6.01
	Fourate	6.22	5.82	6.19	6.65	6.22	6.28	5.88	6.26	6.71	6.28
	Amber 33	6.02	6.18	6.20	6.00	6.10	6.08	6.24	6.27	5.99	6.15
**Mean (B)**	5.79	5.90	5.99	6.01		5.89	5.96	6.06	6.06		
LSD at 0.05 (A)			0.40						0.37		
LSD at 0.05 (B)			Ns						ns		
LSD at 0.05 (AB)			0.58						0.57		

*ns: no significant difference at 0.05 level of probability*.

**Table 5:** Kernel quality traits of five rice cultivars affected by the concentration of Zn during the 2018 and 2019 seasons.

**Attributes**		**Season 2018**					**Season 2019**				
	**(A) Cultivars**	**(B) Foliar application of Zn (mg/L water)**				**Mean (A)**	**(B) Foliar application of Zn (mg/L water)**				**Mean (A)**
		**Water**	**1,500**	**2,000**	**2,500**		**water**	**1,500**	**2,000**	**2,500**	
**Grain thickness**	Sakha 101	1.70	1.57	1.75	2.05	1.77	1.73	1.62	1.74	1.98	1.77
	Giza 178	1.63	1.67	1.66	1.70	1.67	1.62	1.66	1.79	1.97	1.76
	Yasmeen	1.71	1.93	1.62	1.75	1.75	1.74	1.92	1.61	1.85	1.78
	Fourate	1.64	1.69	1.86	2.06	1.80	1.64	1.68	1.82	2.05	1.80
	Amber 33	1.74	1.61	1.61	1.83	1.70	1.73	1.60	1.60	1.92	1.71
Mean (B)		1.68	1.69	1.69	1.87		1.69	1.70	1.71	1.95	
LSD at 0.05 (A)			Ns					ns			
LSD at 0.05 (B)			0.13					0.12			
LSD at 0.05 (AB)			0.30					0.28			
**Grain shape**	Sakha 101	1.74	2.41	2.42	2.01	2.15	1.72	2.39	2.40	1.99	2.13
	Giza 178	2.54	2.54	2.02	2.56	2.42	2.52	2.52	2.00	2.54	2.40
	Yasmeen	1.63	2.56	2.41	3.10	2.43	1.66	2.54	2.60	3.08	2.47
	Fourate	1.57	2.01	2.13	2.97	2.17	1.59	1.93	2.11	3.05	2.17
	Amber 33	1.53	2.43	2.16	2.45	2.14	1.59	2.34	2.47	2.66	2.27
	Mean (B)	1.80	2.39	2.23	2.62		1.82	2.34	2.32	2.66	
LSD at 0.05 (A)			0.22					0.31			
LSD at 0.05 (B)			0.31					0.34			
LSD at 0.05 (AB)			0.71					0.76			
**Amylose content (%)**	Sakha 101	18.86	21.84	22.33	25.03	22.02	18.71	21.69	22.23	25.95	22.15
	Giza 178	18.32	20.12	21.67	25.83	21.49	19.28	19.76	21.52	25.73	21.57
	Yasmeen	17.00	22.05	21.16	29.33	22.39	16.80	21.90	21.01	28.90	22.15
	Fourate	17.67	17.37	23.40	26.67	21.28	18.67	17.27	19.65	26.33	20.48
	Amber 33	15.33	19.67	21.00	22.02	19.51	15.67	20.00	20.28	21.87	19.46
	Mean (B)	17.44	20.21	21.91	25.78		17.83	20.12	20.94	25.76	
LSD at 0.05 (A)			2.15					2.03			
LSD at 0.05 (B)			2.62					2.26			
LSD at 0.05 (AB)			5.85					5.05			
**Gel consistency (GC)**	Sakha 101	82.67	88.50	91.31	92.25	88.68	81.00	82.50	88.91	91.33	85.94
	Giza 178	45.87	56.00	81.83	86.98	67.67	39.33	57.33	74.17	88.67	64.88
	Yasmeen	49.33	63.00	74.00	81.60	66.98	54.67	55.00	70.33	75.54	63.89
	Fourate	83.24	78.07	79.11	80.67	80.27	75.24	71.41	71.11	77.33	73.77
	Amber 33	36.67	69.17	77.98	75.67	64.87	35.67	61.50	69.98	76.67	60.96
	Mean	59.56	70.95	80.85	83.43		57.18	65.55	74.90	81.91	
LSD at 0.05 (A)			5.48					1.67			
LSD at 0.05 (B)			4.60					3.89			
LSD at 0.05 (AB)			10.28					8.68			
**Gelatinization temperature (GT)**	Sakha 101	4.30	4.46	4.88	5.13	4.69	4.20	4.36	4.78	5.03	4.59
	Giza 178	3.33	4.27	4.31	4.79	4.18	3.27	4.17	4.21	4.72	4.09
	Yasmeen	3.00	3.44	3.94	4.15	3.63	3.47	3.34	3.84	4.05	3.68
	Fourate	3.33	4.05	3.61	3.73	3.68	3.70	3.95	3.90	4.19	3.94
	Amber 33	2.30	3.27	3.69	3.68	3.24	2.93	3.27	4.05	3.57	3.46
Mean (B)	3.25	3.90	4.09	4.30		3.51	3.82	4.16	4.31		
LSD at 0.05 (A)			0.35					0.14			
LSD at 0.05 (B)			0.36					0.30			
LSD at 0.05 (AB)		0.81					0.67				

*ns: no significant difference at 0.05 level of probability*.

In our study, the results suggested that foliar application of Zn improved grain yield and quality of rice, because Zn released nutrients gently and gradually during critical growth stages. Also the foliar application of Zn is more easily, quickly absorbed and assimilated in plants (Yuvaraj and Subramanian, 2014), the application of nutritional components such as Zn ran to enhanced panicle length (Kandil et al., 2020a; Wang et al., 2022).

Zinc is one of the most important microelements for the rice and its deficiency may cause a significant reduction in the rice yield in flooding lands (Smith and Hamel, 2012). The foliar use of Zn may have enhanced the growth and increased the number of fertile tillers and filled grains per panicle number in rice increased grain yield and its components by increasing absorption and the allocation of other vital nutrients in the plant, thus enhancing the metabolic processes of the plant (Naik and Das, 2007; El-Naggar et al., 2020; Gomaa et al., 2021). In this study, the increments might result from the important role of Zn in many biochemical reactions within the plants. Zn modifies and/or regulates the activity of carbonic anhydrase, an enzyme that regulates the conversion of carbon dioxide to reactive bicarbonate species for fixation to carbohydrates in these plants. In addition, Zn also, a part of other numerous enzymes such as superoxide dismutase and catalyze, prevents oxidative stress in plant cells (Shehata et al., 2009). Similar results are compared to those reported by Slaton et al. (2005) who detected that applying Zn at 45 days after planting has the most correlation (*r* = 0.89^**^) with the rice yield. The use of Zn increases the distribution of nutrients to the plant's generative organs, such as panicles, and increases total dry matter accumulation (Amanullah and Inamullah, 2016). Also, an increase in the previously mentioned characteristics increased the grain yield of rice where the foliar application of Zn increased the grain yield by ~30% compared with the control treatment (Farooq et al., 2018); however, adding Zn significantly increases the grain yield of rice (Jaksomsak et al., 2018). Another similar result showed that three times foliar application of Zn recorded the maximum values of panicle length, spikelets numbers/panicles, panicle weight, filled grains percent, and filled grains numbers/panicle. Panicle number/m2 and 1,000-grain weight were slightly affected by the number of zinc foliar applications. The application of zinc foliar three times at the level of 600 ppm exhibited the highest values of grain, straw, biological, and Grain crude protein (GCP) yields as well as most of the yield attributes and nitrogen physiological parameter (Fergany, 2018). Also, the application of Zn improves the protein content (%) and the quality of rice through amino acid accumulation and protein synthesis due to increased N metabolism (Bashir et al., 2011). Additionally, Jaksomsak et al. (2018) and Prasad et al. (2013) found that Zn foliar spray significantly increased Zn accumulation in all unpolished rice cultivars. Foliar Zn fertilization is the most economical means of alleviating its deficiency in rice and improving the productivity and grain Zn concentration, thus contributing to higher nutritional value (Verma et al., 2018). The following parameters were significantly increased by the use of Zn from different sources: hulling, milling, head rice recovery, grain length, grain breadth, grain length after cooking (KLaC), volume expansion ratio, water uptake, amylose content, alkali spreading value, protein content, and protein yield (Verma et al., 2018). In addition, Zn is also known for its critical role in controlling gene transcription because of its role in the Zn finger proteins that regulate gene transcription and the coordination of other biological processes by DNA-binding Zn-finger motifs (Rhodes and Klug, 1993). The present results are in the same line as those of Phattarakul et al. (2012) who suggested that promoting the grain Zn content by foliar Zn application results in Zn being available for re-translocation into grains. One of the reasons for the stimulated transport of Zn into grain after the flowering stage might be related to significant increases in protein biosynthesis during the early stage of seed formation (Martre et al., 2003). As discussed by Ozturk et al. (2006) and Cakmak et al. (2010), increasing grain protein concentrations creates a sink for Zn, and there is a close positive correlation between grain protein and Zn concentrations. It means that enhancing grain yield was accompanied by an increase in straw yield by the same trend. In this condition and according to the achieved results, foliar application of Zn can be substituted by soil application of Zn fertilizer in rice and leads to improve the yield of rice (Rahman et al., 2008).

### Cytological Characteristics

**Figure 6:**
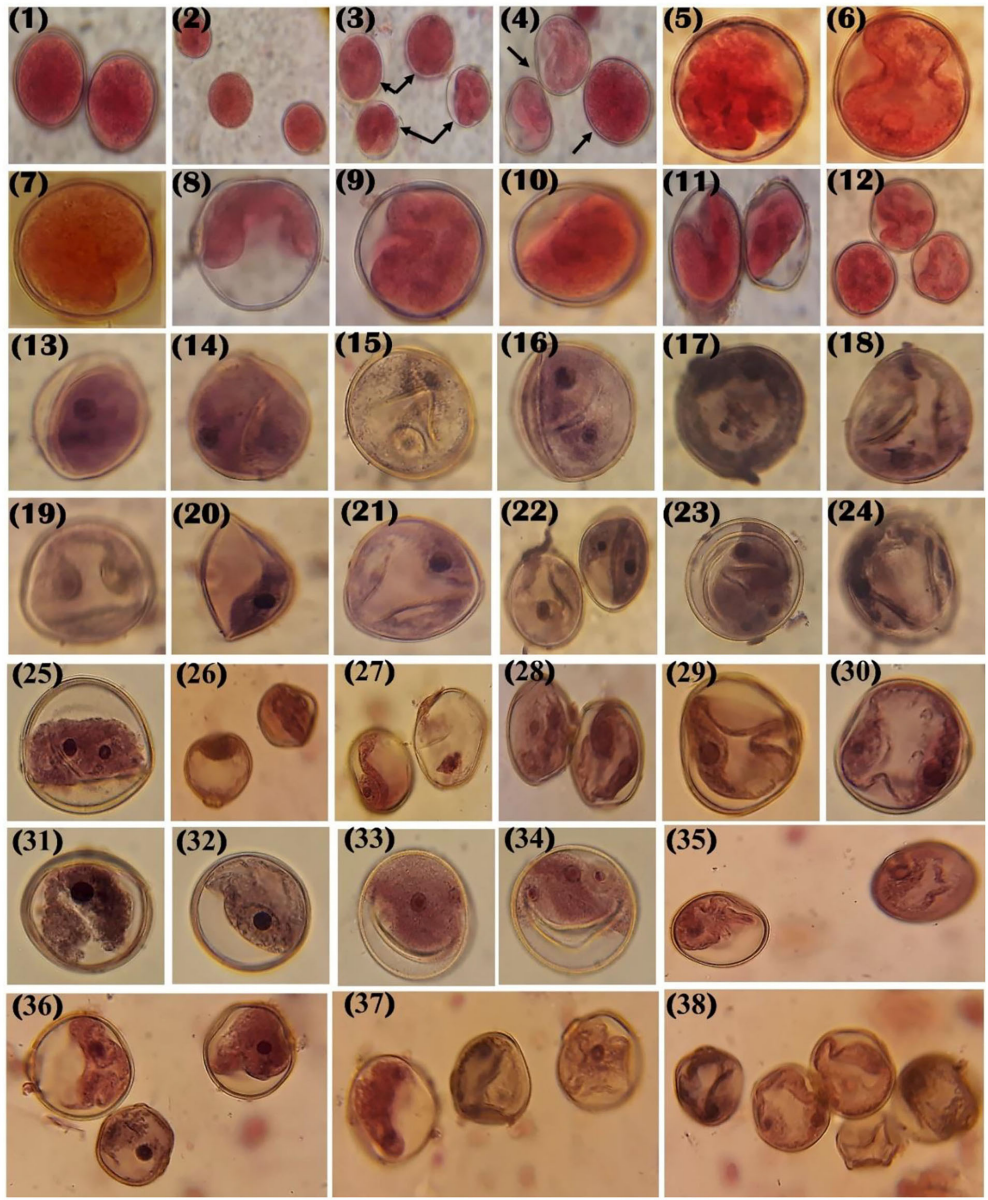
Effect of Zn concentration (1,500, 2,000, and 2,500 mg/L) on pollen viability in rice cultivars (1–4) control showing completely fertile pollen grains (arrowheads) with full stanning and shape filled with normally stained starch in the rice cultivars Sakha1010, Skha178, Fourate, Yassimen, Amaber; (3–4) showing the difference between completely fertile and semifertile pollen grains in control; (5–12) pollens with stickiness, ultrastructural variations in the cell walls of the pollen grain, increase in ultrastructural variations (~15:30%) in the exterior and interior walls, non-stained pollen grains with different aberrations such as stickiness, shrinkingly in pollen content with a large gap in capacity; (13–38) pollen grains with brown or light tan color (waxy); incompletely or fully degenerated content, and light red color and nearly 50:70% decrease in cytoplasm content, light red color and death of some parts in pollen grains, stickiness, and a big gap in pollen capacity.

This investigation was conducted to examine the impact of Zn fertilizers on pollen grain viability and aberrations percentage in rice (*Oryza sativa* L.). Data in Table 6 and Figure 6 show the impact of Zn fertilizers on pollen grain viability and structure of rice (*O. sativa*) cultivars from Egypt and Iraq. The results showed that total observed pollen grains and viability % in Sakha 101 cultivar under Zn fertilizers concentrations ranged from 1,400 to 1,435 cells in the average of 1,429.25 cells compared with control which was 1,452 cells. About 2,500 mg/L of Zn showed that a high percentage of aberration was 26.69% compared with control which was 13.29% (Table 6). With the increase in Zn fertilizers, the results showed a decrease in viability percentage that was 86.71 (under control) to 73.81% under the high level. While in Sakha 178, the values of total examined cells were from 1,400 to 1,412 cells and 1,398 control, the shrunken pollen grains increased from 35 to 121 cells under 2,500 mg/L and the percentage of aberration was high at the highest level of 25.28%. Control plants showed the lowest aberrations percentage for all rice cultivars and thus ranged from 9.42 (Yassimen) to 28.19% (Fourate), while under the highest level of Zn fertilizers, the viability percentage decreased to 71.81% (Fourate).

**Table 6:** Total observed pollen grain, unstained pollen, shrunken pollen, fertile pollen, viability percentage, and aberrations percentage of pollen grain of rice (*O. sativa*) in response to the different concentrations of Zn fertilizers.

**Cultivars**	**Zn (mg/L)**	**Observed pollens**	**Unstained pollens**	**Shrunken pollens**	**Sterile pollens**	**Fertile pollens**	**Viability %**	**Aberration %**
Sakha 101	Control	1,452	128	43	22	1,259	86.71	13.29
	1,500	1,430	126	53	41	1,210	84.62	15.38
	2,000	1,400	140	52	48	1,160	82.86	17.14
	2,500	1,435	153	75	155	1,052	73.31	26.69
	average	1429.25	136.75	55.75	66.5	1170.25	81.87	18.13
	SD	21.65	12.47	13.60	60.01	88.59	5.92	5.92
Sakha 178	Control	1,398	94	35	42	1,227	87.77	12.23
	1,500	1,400	67	43	55	1,235	88.21	11.79
	2,000	1,400	67	53	93	1,187	84.79	15.21
	2,500	1,412	101	121	135	1,055	74.72	25.28
	average	1402.5	77.25	70.5	78.75	1,176	83.87	16.13
	SD	6.40	16.17	34.85	45.15	83.35	6.29	6.29
Yassimen	Control	1,380	45	43	42	1,250	90.58	9.42
	1,500	1,440	66	80	84	1,210	84.03	15.97
	2,000	1,400	74	85	101	1,140	81.43	18.57
	2,500	1,450	112	101	101	1,136	78.34	21.66
	average	1417.5	74.25	77.25	82	1,184	83.60	16.40
	SD	33.04	27.98	24.53	27.84	55.59	5.20	5.20
Amaber 33	Control	1,351	60	54	87	1,150	85.12	14.88
	1,500	1,430	103	93	122	1,112	77.76	22.24
	2,000	1,406	77	112	124	1,093	77.74	22.26
	2,500	1,412	102	143	152	1,015	71.88	28.12
	average	1399.75	85.5	100.5	121.25	1092.5	78.13	21.87
	SD	34.06	20.82	37.22	26.63	56.84	5.42	5.42
Fourate	Control	1,411	67	55	39	1,250	88.59	11.41
	1,500	1,395	154	124	114	1,003	71.90	28.10
	2,000	1,422	138	148	132	1,004	70.60	29.40
	2,500	1,412	133	120	145	1,014	71.81	28.19
	average	1,410	123	111.75	107.5	1067.75	75.73	24.27
	SD	11.17	38.39	39.80	47.40	121.60	8.60	8.60

Data in Figure 6 showed the control pollen grain cells with the increase in pollen viability, the cytoplasm is densely distributed, and many round-like units were found, with dark red color and full capacity, and the wall is round without empty spaces. Also, the figure showed the effect of Zn on different changes, such as the stanning color and warp in the cell wall (not round), stickiness in cytoplasm, and increase in ultrastructural changes; in addition, there are some structural variations in the exine and interior cell walls of the pollen grain and also growth in the structural differences (more than 10%) in the exine and interior cell wall of the pollen grain, stickiness in cytoplasm content. Effect of commercial Zn on rice cultivar (Yasmeen) showed shrunken pollen with gap in pollen capacity and stickiness in cytoplasm, and finally, for Anbar cultivar showing stickiness in cytoplasm and whole space between the exine and interior walls. Data in Figure 6 show the effect of Zn fertilizers, on the structural changes in pollen grains, which increased in the structural variations (more than 25%), for Sakha 178 which showed a decrease in pollen size and stickiness in the cytoplasm with shrunken in pollen walls. Yasmeen cultivar shows damage in pollen content with stickiness in the cytoplasm with shrunken pollen walls, cytoplasm stickiness, damage to the cell wall of the pollen grain, and a big gap in pollen capacity.

The recent results are in agreement with this finding which stated that the pollen morphology has become an essential descriptor (Lechowicz et al., 2020; Souza et al., 2022), and the pollen grains have their own unique set of attributes such as the size, exine structure, and pore size (Evrenosoglu and Misirli, 2009). This study considers a helpful tool to monitor the impact of Zn fertilizers on pollen grain viability test for understanding the viability and capacity of pollen grain which is vital for the reproductive biology and genetic breeding of plants, and this is in agreement with the study of Dane and Lang (2004) and Gomaa et al. (2021). Pollen staining tests are among the most reliable and most widely used pollen viability tests (Pierson and Cresti, 1992). Also, these results (Abdelsalam et al., 2021) suggested that the findings of pollen germination and tube growth are essential for knowledge fertilization and seed development in flowering plants. Our results suggested that there is a high effect of mineral fertilizers on rice pollen grain and these findings are in agreement with the study of Stanley and Linskens (1974) who detected that there are many factors that can have an effect on the pollen germination; one of them is the application of fertilizers. Our results indicated that there is a genotoxic effect of using microparticles in fertilizers, which cause aberration in pollen grain structure and fertility, and these results in the same trend with Lienert et al. (2011) reported the use of these materials and their numerous potential routes of entry into the environment, and they assumed pollen mortality and inhibition on germination. Finally, many studies detected the side effect of using mineral materials on organisms and their toxicities (Benzon et al., 2015; Abdelsalam et al., 2019; Mosa et al., 2021; Silva et al., 2022). Other studies (Hegazi et al., 2010) suggested using bio-fertilizers that contain different strains has resulted in a reduction in the use of chemical fertilizer and provides high-quality products. On the other hand, the study of Georgieva et al. (2017), Fouda et al. (2020), and Kandil et al. (2020b) revealed a positive impact of the application of organics on *in vitro* pollen germination and tube elongation in *P. sativum*, and they reported that the obtained results enrich the current information about the activity of s and they recommended in the future to make more detailed research to clarify the mechanism of action and the consequences of using s. Also, Fry et al. (2012) stated that the size of Fe and Mg particles had a positive impact on photosynthesis, and improving the photosynthesis could provide high soluble sugar in the anther wall that will increase pollen viability.

## Conclusion

This study aimed to provide the most suitable rice variety performance and the Zn, by comparing the effects of different varieties and foliar Zn spray on rice yield, yield component, and quality. The studied varieties differed in all the characteristics; Zinc application rates significantly increased the rice yield parameters, straw, and grain yields over control. The highest Zn rates of 2,500 mg/L resulted as better treatment followed by 2,000 mg/L, and Giza 168, as well as Sakha 101 cultivars, gave higher performance under these rates in yield, yield component, and kernel quality trait. The obtained results of this study would be beneficial to mitigate Zn deficiency in rice and therefore improve zinc use efficiency. Also, this study revealed that the commercial Zn in high concentration was genotoxic to pollen grain and caused abnormalities in their structure and function. Thus, it is necessary to formulate a suitable rate of foliar Zn spray that does not show genotoxicity or produce other damage or aberrations in pollen grains.

## Data Availability Statement

The raw data supporting the conclusions of this article will be made available by the authors, without undue reservation.

## Author Contributions

EK, AE-B, and NA: data curation. EK, AE-B, DT, MM, AAA-H, and RG: formal analysis. DT: investigation. EK, AE-B, MM, and NA: methodology. EK, AE-B, and RG: resources. AE-B, DT, and AAA-H: software. EK, RG, AAA-H, HA, JJ, and NA: writing—original draft. NA, AAA-H, JJ, and HA: writing, reviewing, and editing. AAA-H and HA: funding. All authors have read and agreed to the published version of the manuscript.

## Funding

This research was funded by the Deanship of Scientific Research, King Saud University, through the Vice Deanship of Scientific Research Chairs.

## Conflict of Interest

The authors declare that the research was conducted in the absence of any commercial or financial relationships that could be construed as a potential conflict of interest.

## Publisher's Note

All claims expressed in this article are solely those of the authors and do not necessarily represent those of their affiliated organizations, or those of the publisher, the editors and the reviewers. Any product that may be evaluated in this article, or claim that may be made by its manufacturer, is not guaranteed or endorsed by the publisher.
